# Machine learning-assisted non-destructive plasticizer identification and quantification in historical PVC objects based on IR spectroscopy

**DOI:** 10.1038/s41598-022-08862-1

**Published:** 2022-03-23

**Authors:** Tjaša Rijavec, David Ribar, Jernej Markelj, Matija Strlič, Irena Kralj Cigić

**Affiliations:** 1 Faculty of Chemistry and Chemical Technology, University of Ljubljana, Ljubljana, Slovenia; 2 Institute for Sustainable Heritage, University College London, London, UK; 3 Museum Conservation Institute, Smithsonian Institution, Suitland, MD USA

**Keywords:** Analytical chemistry, Infrared spectroscopy, Polymer chemistry, Computational methods

## Abstract

Non-destructive spectroscopic analysis combined with machine learning rapidly provides information on the identity and content of plasticizers in PVC objects of heritage value. For the first time, a large and diverse collection of more than 100 PVC objects in different degradation stages and of diverse chemical compositions was analysed by chromatographic and spectroscopic techniques to create a dataset used to construct classification and regression models. Accounting for this variety makes the model more robust and reliable for the analysis of objects in museum collections. Six different machine learning classification algorithms were compared to determine the algorithm with the highest classification accuracy of the most common plasticizers, based solely on the spectroscopic data. A classification model capable of the identification of di(2-ethylhexyl) phthalate, di(2-ethylhexyl) terephthalate, diisononyl phthalate, diisodecyl phthalate, a mixture of diisononyl phthalate and diisodecyl phthalate, and unplasticized PVC was constructed. Additionally, regression models for quantification of di(2-ethylhexyl) phthalate and di(2-ethylhexyl) terephthalate in PVC were built. This study of real-life objects demonstrates that classification and quantification of plasticizers in a general collection of degraded PVC objects is possible, providing valuable data to collection managers.

## Introduction

Poly(vinyl chloride) (PVC) is a modern polymeric material and one of the most widely used plastics today^1^. Since its commercialization, it has been used in a variety of applications, including clothing, toys, tubing and piping, flooring, and medical devices^2,3^. Plasticizers are used to improve and adjust the mechanical properties of the material by increasing flexibility, reducing viscosity, and decreasing friction during manufacture. Plasticizers can account for up to 50% of the total mass of PVC objects^4–6^. Because of its versatility PVC has also been used as a material for artworks^4,7,8^ and represents a significant part of modern and contemporary collections^9^. The challenges of preserving PVC are well recognised in the literature^10–12^, and some are related to the plasticizers in the material^13^. There are reported cases of plasticizer accumulating on the surface, which can trap dust and dirt, as well as plasticizer loss, which can cause warping, cracking, and structural failure^7,13–15^. Phthalates, terephthalates, adipates, trimellitates, and citrates represent the majority of commercially-used plasticizers. Dibutyl phthalate (DBP), diisobutyl phthalate (DIBP), di(2-ethylhexyl) phthalate (DEHP), diisononyl phthalate (DINP), diisodecyl phthalate (DIDP), di(2-ethylhexyl) terephthalate (DOTP), di(2-propylheptyl) phthalate (DPHP), and diisononyl cyclohexane-1,2-dicarboxylate (DINCH) were found as the most interesting plasticizers based on their properties, historic significance and market popularity (Supplementary Fig. S1). Market popularity has shifted over the decades mainly due to legislation changes in Europe and the USA. DBP and DIBP are short-chain phthalate plasticizers that have been primarily used in combination with DEHP and DINP^16^. DBP is a suspected endocrine disruptor, so its usage is regulated and restricted for use in toys in the EU^17^. DEHP had been used as a general-purpose plasticizer for all consumer products, including toys, food contact material, cosmetics, medical devices and household products. In 2008, it was classified as hazardous in Europe^18^ and the USA, and was replaced by higher molecular weight phthalates (DINP and DIDP) as general-purpose plasticizers. DOTP is a terephthalate isomer of DEHP, with similar plasticization abilities, but is subject to less regulatory restrictions and pressure due to reduced toxicity compared to traditional *ortho*-phthalate esters. DINCH is the hydrogenated form of DINP, which is used in Europe since 2006 as a plasticizer for PVC in sensitive products, such as medical devices, toys, and food packaging^19^. Based on these general market trends, plasticizer identification could also serve as a rough estimate for the age of objects with unknown histories.

Loss of plasticizers in PVC is a significant process during an object’s lifetime. It can occur by evaporation into the surrounding air, extraction into liquids, or migration into another polymeric material. The properties of the plasticizer, such as molecular weight, vapour pressure, polarity and compatibility with the polymer, affect its migration rate^3^. In general, the loss of low molecular weight plasticizers is greater than that of high molecular weight plasticizers^20^. The diffusion rate of DEHP was found to be higher than that of DOTP^21^, which could be due to better structural compatibility with the PVC polymer. Additionally, the role of identity and content of plasticizers in the degradation of PVC objects is still not well-understood. Sources report that certain plasticizers promote degradation, while others slow it down^16^. Therefore, in the effort to preserve historically valuable PVC collections, conservators need a readily available method to identify and quantify plasticizers.

Identification and quantification of plasticizers can be achieved by different analytical techniques. Most protocols involve the removal of additives from the polymer by solvent extraction or dissolution prior to identification by spectroscopy or chromatography^22–30^. There are also some non-destructive sampling techniques that can be used with GC–MS for identification, e.g. using SPME fibers, active and passive samplers. Some historical PVC objects exhibit surface accumulation of phthalates, which can be sampled by swabbing^13^. Spectroscopic techniques, such as infrared and Raman spectroscopy can provide some insight into plasticizer identification. Attenuated total reflectance (ATR) IR spectroscopy can be used to collect high-quality spectra, but it requires good contact between the sample and the ATR crystal^31^. Therefore, very brittle samples and objects of unsuitable geometry cannot be analysed non-destructively. Even good quality spectra are unreliable for unambiguous identification by the assignation of characteristic vibrational bands because different plasticizer types absorb in the same spectral region and may overlap due to common functional groups. In addition, the similarities of di-alkyl phthalate spectra within plasticizer groups are high. Manual peak assignment and interpretation of spectra is further complicated due to the shifting of polymer-characteristic peaks^32–34^. Interactions between polymer and plasticizer can impact vibrational energies of functional groups in the polymers and cause a shift. Research by Berg et al.^35^ reported that FT-Raman spectroscopy can be used for direct determination of phthalate plasticizers in PVC based on the intensities of characteristic peaks, but differentiation of homologues is difficult. The technique was also unsuitable for the determination of adipate esters^36^.

The main limitations of commonly used instrumental analysis are destructive sampling and specialized instrumentation not available to most museums or general laboratories. Due to these limitations, attempts have been made to obtain the same information by combining non-destructive techniques, which are ideally available as portable instruments, with advanced machine learning methods. Information of chemical nature, such as the identity and content of plasticizers in PVC objects, can aid in planning the long-term conservation of cultural heritage objects.

Multivariate analysis (MVA) has been used in a variety of heritage science and analytical applications because of its ability to analyse non-linear relationships, flexibility, and speed of data processing. MVA can be used to obtain qualitative and quantitative information from spectral data of polymeric materials. It is used for the classification of polymers with NIR spectroscopy and for predicting the content of additives. Some MVA approaches have already been implemented in NIR and mid-IR software and are available for specific IR instruments^37,38^. FT-NIR was combined with partial least squares (PLS) to develop a calibration plot used for the determination of DEHP plasticizer in PVC^37^. Phthalates can be determined down to 0.1% total content with a transmission FTIR spectrometer by pressing a sample to 0.5 mm thickness. The application could not distinguish between phthalate plasticizers^35^. Independent component analysis (ICA) was combined with ATR-MIR spectroscopy for the identification and quantification of plasticizers in polylactide (PLA)^39^. The methodology appears promising, although its applicability on objects not included in the modelling was not tested.

Non-destructive methods of analysis are frequently required in the field of heritage science. NIR spectroscopy was combined with PLS analysis to date fiber-based gelatine silver photographic papers^40^. Genetic algorithms were used to determine gelatine content in historical papers based on FTIR and NIR spectroscopy^41^. Micro-Raman spectroscopy and PLS regression were used to classify iron-based inks of historical papers and quantify organic acids^42^. Modelling was already used to predict the degradation of unstable historical polymers^43^.

Previous studies on the identification and quantification of plasticizer content in PVC objects demonstrate that such information can be obtained using non-destructive methods^44^. No such studies have been reported for the analysis of plastic objects in the field of heritage science where accessible options in the form of application notes are often of limited use because they rely on purpose-made samples and are constrained to a specific plasticizer type or sample size. Museum objects may have undergone changes due to degradation, which were not accounted for in computation modelling^37,38^. Furthermore, heritage PVC collections remain poorly characterized and surveyed, and the results presented here provide a first insight into the prevalence and representation of plasticizers. The study presented here, using more than a hundred real-life objects, demonstrates that classification and quantification of certain plasticizers based on such collections are possible. The objects vary in plasticizer type and content, thickness, fillers, stabilizers and other additives, degradation stage and storage history, so they are considered representative of objects in heritage collections. Experimental data was obtained from analysing samples in the collection. A classification model capable of identifying DEHP, DOTP, DINP, DIDP, a mixture of DINP with DIDP, and unplasticized PVC from ATR-FTIR or NIR spectroscopy was built. In addition, a regression model for quantifying DEHP and DOTP in PVC objects was also built. Since near-infrared light can penetrate much farther than mid-infrared light, mid-IR spectra reveal surface information about the objects, while NIR spectra reveal bulk information. Considering that surface concentration is proportional to bulk concentration, the differences in penetration depth were considered inconsequential for building machine learning models.

Machine learning (ML) algorithms are used to make predictions or decision models based on training data, using a number of different approaches, from simple to sophisticated. As proof of concept, we used six common but distinctly different approaches and compared their efficacy in solving the problem of identification and quantification of plasticizers in PVC. Linear discriminant analysis (LDA) and naïve Bayes classification (NBC) were used as the simplest approaches. Support vector machines (SVM), k-nearest neighbours (kNN), decisions trees (DT) and extreme gradient boosted decision trees (XGBDT) are more advanced methods, requiring hyperparameter tunning. Cutting edge algorithms, such as deep-learning have also been recently used with success^45–48^. Advances in unsupervised learning with encoding edges could further improve classification of challenging studies^49^.

## Results and discussion

### Identification and quantification of plasticizers by gas chromatography

Plasticizers in PVC objects were identified by GC–MS in a full-scan screening (Supplementary Fig. S1). Characteristic ions were determined for individual plasticizers (Table 1).

**Table 1:** Selected ions for SIM mode acquisition (GC–MS).

Analyte	Retention time (min)	Quantifying ion (*m/z*)	Qualifying ion (*m/z*)	Qualifying ion (*m/z*)
DBP	6.36	149	223	205
DEHP	8.09	149	167	279
DOTP	8.60	261	167	149
DPHP	8.84	307	149	167
DINCH	8.3–9.0	155	127	281
DINP	8.6–9.4	293	149	127
DIDP	8.9–10.0	307	149	167

All identified plasticizers, with the exception of DIBP, were available as pure compounds that allowed quantitative analysis. Plasticizers in PVC objects that contained DBP, DEHP, DPHP, or DOTP were quantitatively determined using GC-FID (Supplementary Fig. S2). Diisobutyl phthalate (DIBP) was found in only 2 objects and was quantified using DBP as standard, as the flame ionization detector gives a response proportional to the number of carbon atoms. DINCH, DINP and DIDP are commercial plasticizers and are essentially branched isomers. Due to their similar structure, DINCH, DINP and DIDP cannot be identified or quantified by chromatography alone because multiple peaks appear over an extended retention time interval. Using GC–MS, selected peaks were used as quantifying ions, and their identity was confirmed with matching qualifying ions (Table 1)^25^. The total areas of compound-specific ion fragments under specific peaks in the selected time intervals were used for the quantitative determination of DINP, DIDP and DINCH (Supplementary Fig. S3).

**Figure 1:**
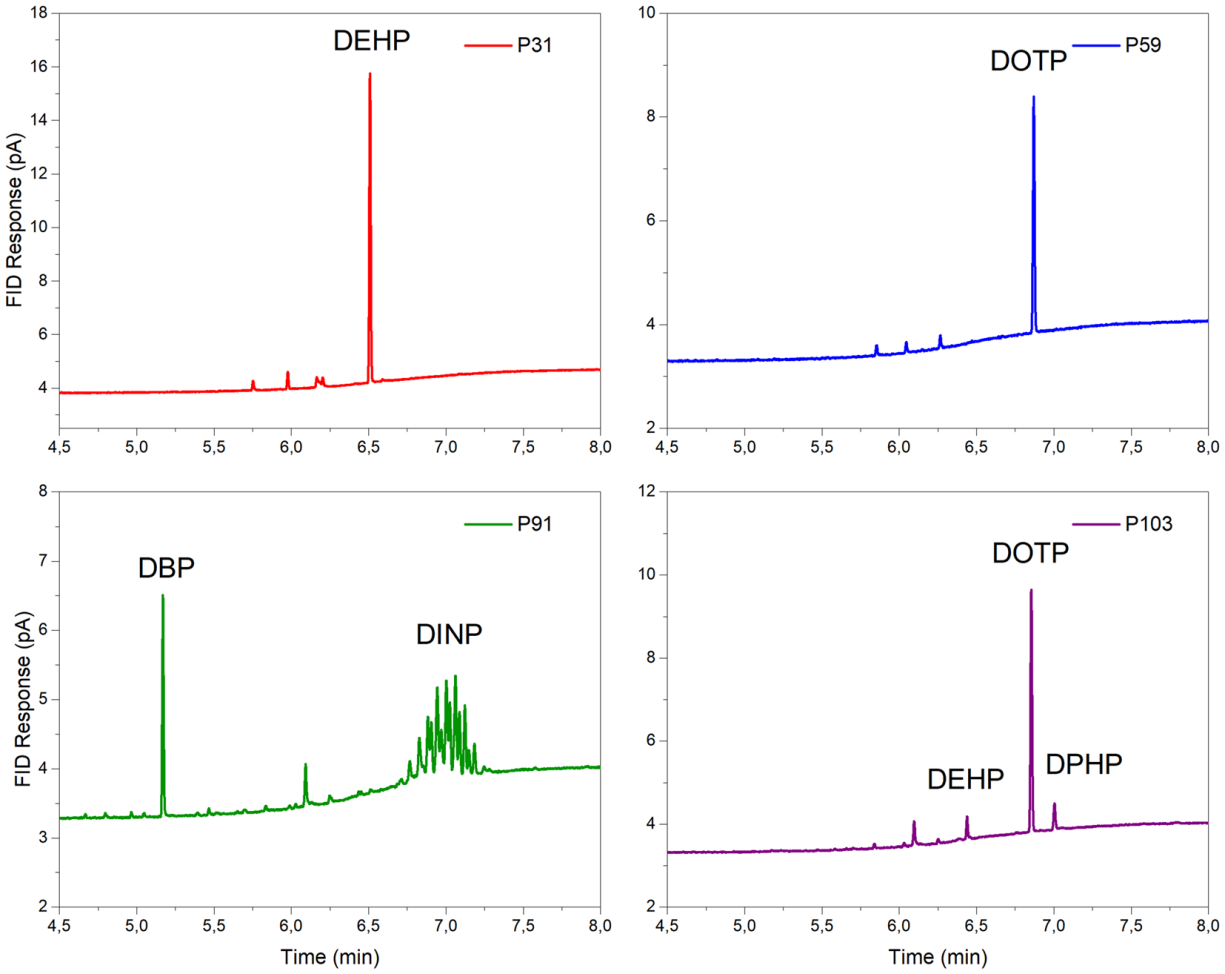
GC-FID chromatograms of hexane solutions of different PVC samples. Sample P31 with DEHP; sample P59 with DOTP, sample P91 with DBP and DINP and sample P103 with DOTP, DPHP and DEHP. Unassigned peaks also occur in blank runs.

**Figure 2:**
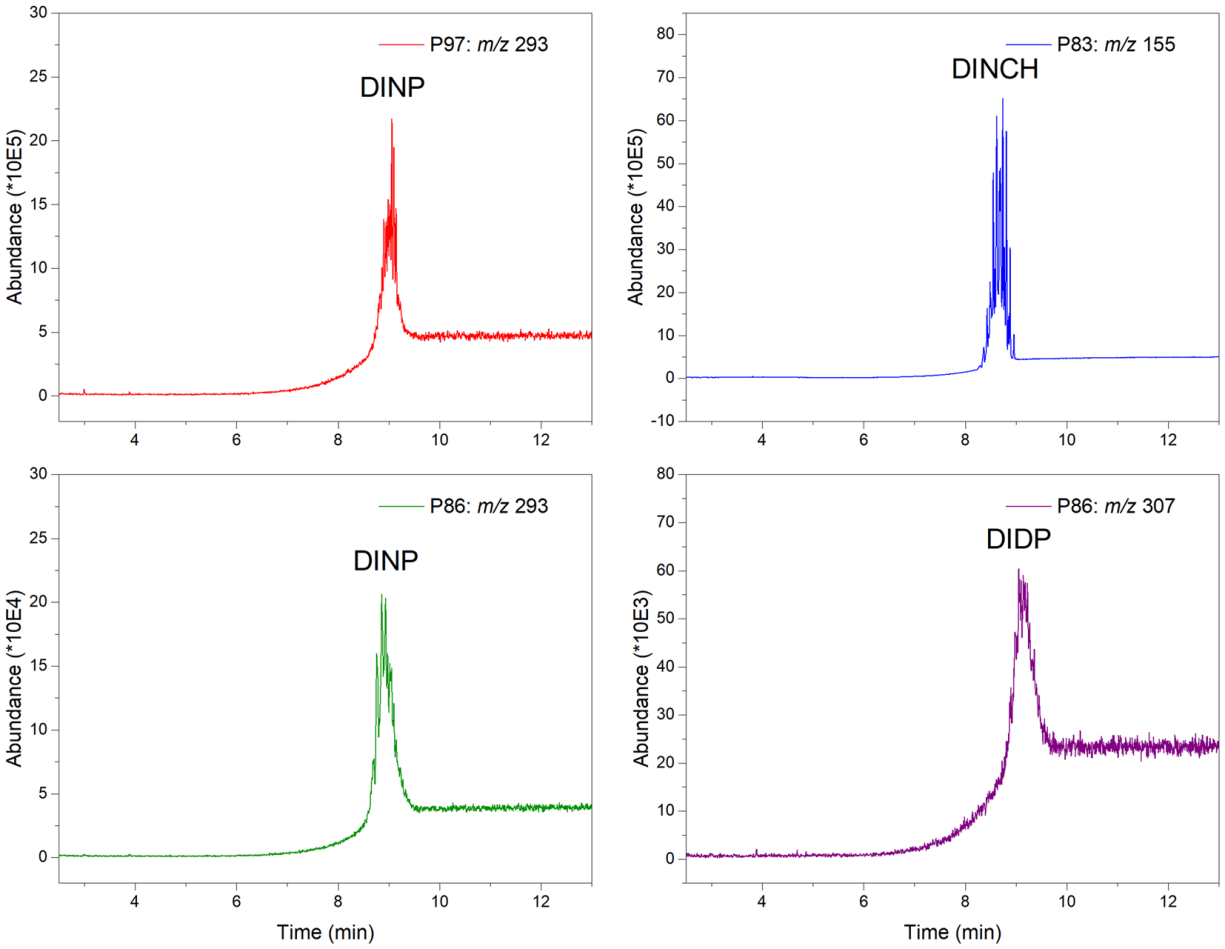
GC–MS chromatograms in SIM mode for plasticizer-specific ion fragments. These are *m/z* 293 for DINP in sample P97 and sample P86, *m/z* 307 for DIDP in sample P86 and *m/z* 155 for DINCH in sample P83.

Certain PVC objects contained 2–3 plasticizers (Fig. 1), which were also analysed by GC–MS (Fig. 2). The bottom two chromatograms in Fig. 2 display *m/z* 293 and *m/z* 307 for the same sample P86, which contains both DINP and DIDP plasticizers, as evident from their respective signals. In Fig. 2, the peak shape for DINP in sample P86 is different from the sample P97, due to the presence of DIDP. Plasticizers were quantified in all samples using linear least-squares regression (all results of the analyses are shown in Supplementary Tables S1 and S2). The parameters of the least-square regression equation for all the analytes for GC-FID and GC–MS analyses are listed in Table 2.

**Table 2:** Parameters of the least-square regression equation of DBP, DEHP, DOTP, DPHP, DINCH, DINP and DIDP.

Analyte	Technique	Linear range (mg/L)	Slope	Intercept	R^2^
DBP/DIBP	GC-FID	10–50	0.0063	0.0016	0.9983
DEHP	GC-FID	10–50	0.0066	0.0064	0.9987
DOTP	GC-FID	10–50	0.0067	0.0035	0.9992
DPHP	GC-FID	10–50	0.0059	0.0057	0.9984
DINCH	GC–MS	5–15	39960	− 59320	0.9980
DINP	GC–MS	5–15	6750	− 7770	0.9992
DIDP	GC–MS	5–15	5920	− 900	0.9986

### General objects statistics

**Figure 3:**
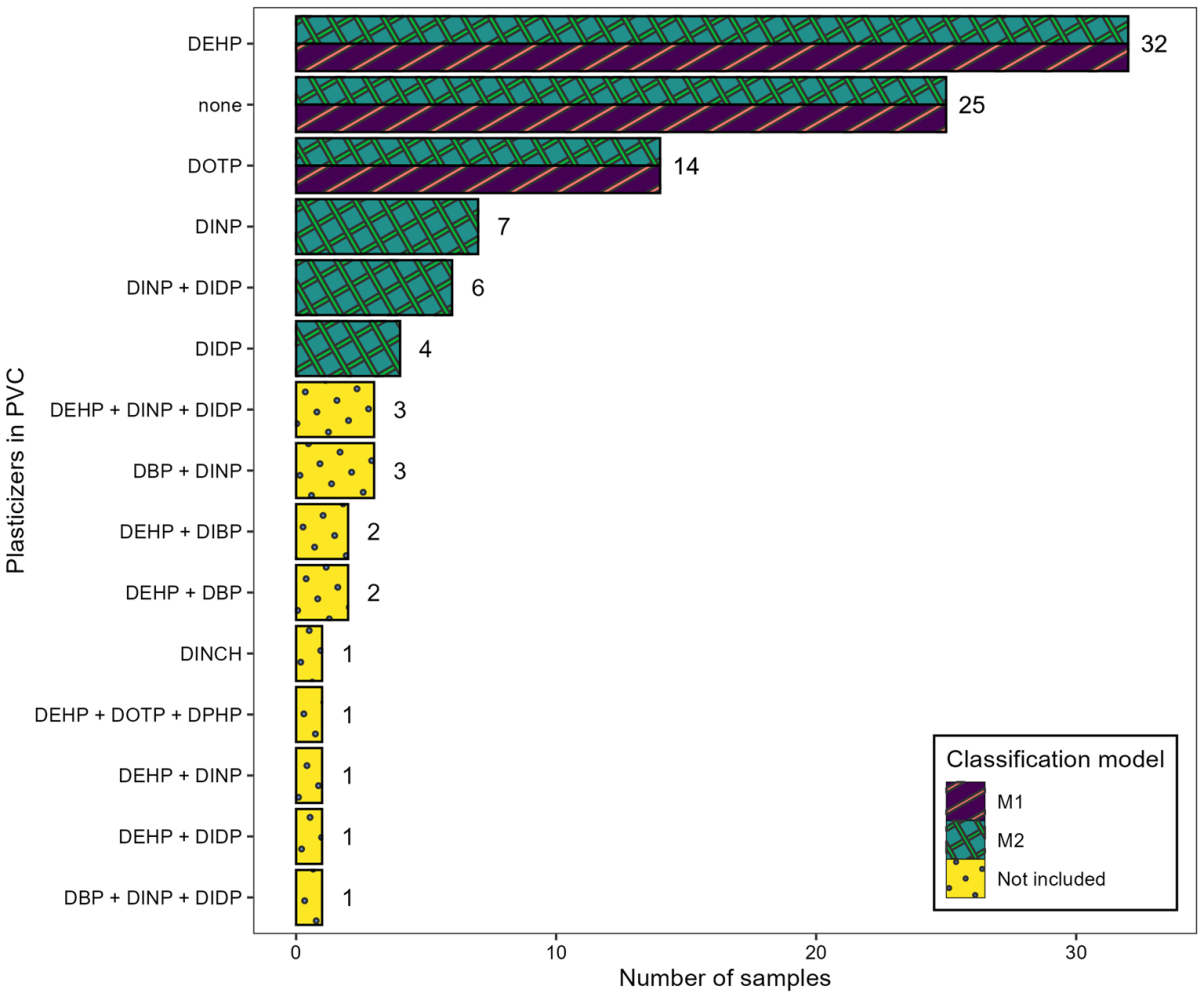
Histogram showing the number of objects per plasticizer or combinations thereof, in the examined PVC collection. Classification models M1 and M2 were developed using objects as indicated in the legend.

The collection of 103 PVC objects was statistically analysed in regard to their plasticizer content. 25 objects contained no plasticizer, 58 objects contained a single plasticizer, and 20 objects contained 2–3 plasticizers (Fig. 3). The complete dataset with plasticizer identification and content is available in Supplementary Table S1. The plasticizer content is almost symmetrically distributed (Supplementary Fig. S4), with a maximum plasticizer content of 47% and a median of 18.1%. However, the distribution for individual plasticizers differs, e.g. DEHP content ranges from 6.7 to 28.9% with a median of 13.3%, whereas DINP content ranges from 15.3 to 40% with a median of 27.0%.

This collection contains 17% of objects with mixed plasticizers, which is a considerable amount and comparable to other surveys^50,51^. The most common mixture of plasticizers was a combination of DINP with DIDP (6 objects). DEHP is frequently combined with different plasticizers, such as DBP, DIBP, DINP or DIDP. The simultaneous presence of three plasticizers, a combination of DEHP, DINP and DIDP, was found in 3 objects; while the combination of DEHP, DOTP and DPHP was found in one object. Machine learning requires an adequate number of objects in a given group^52^. Therefore, two classification methods were developed, one with only highly populated groups, and one including less populated groups, however, groups with 3 objects or less were considered too small. Joining all of these into one larger group seemed ineffective since it would encompass objects with single and mixed combinations of 7 different plasticizers. The classification model M1 was developed using objects with no plasticizers (“none”) and with objects containing either DEHP or DOTP (i.e., groups with N > 7). This model was subsequently expanded into model M2 to include objects with DINP and/or DIDP, i.e., with a single plasticizer or a mixture of both was identified. PLS regression development was possible for objects containing DEHP or DOTP.

### Classification algorithms

**Figure 4:**
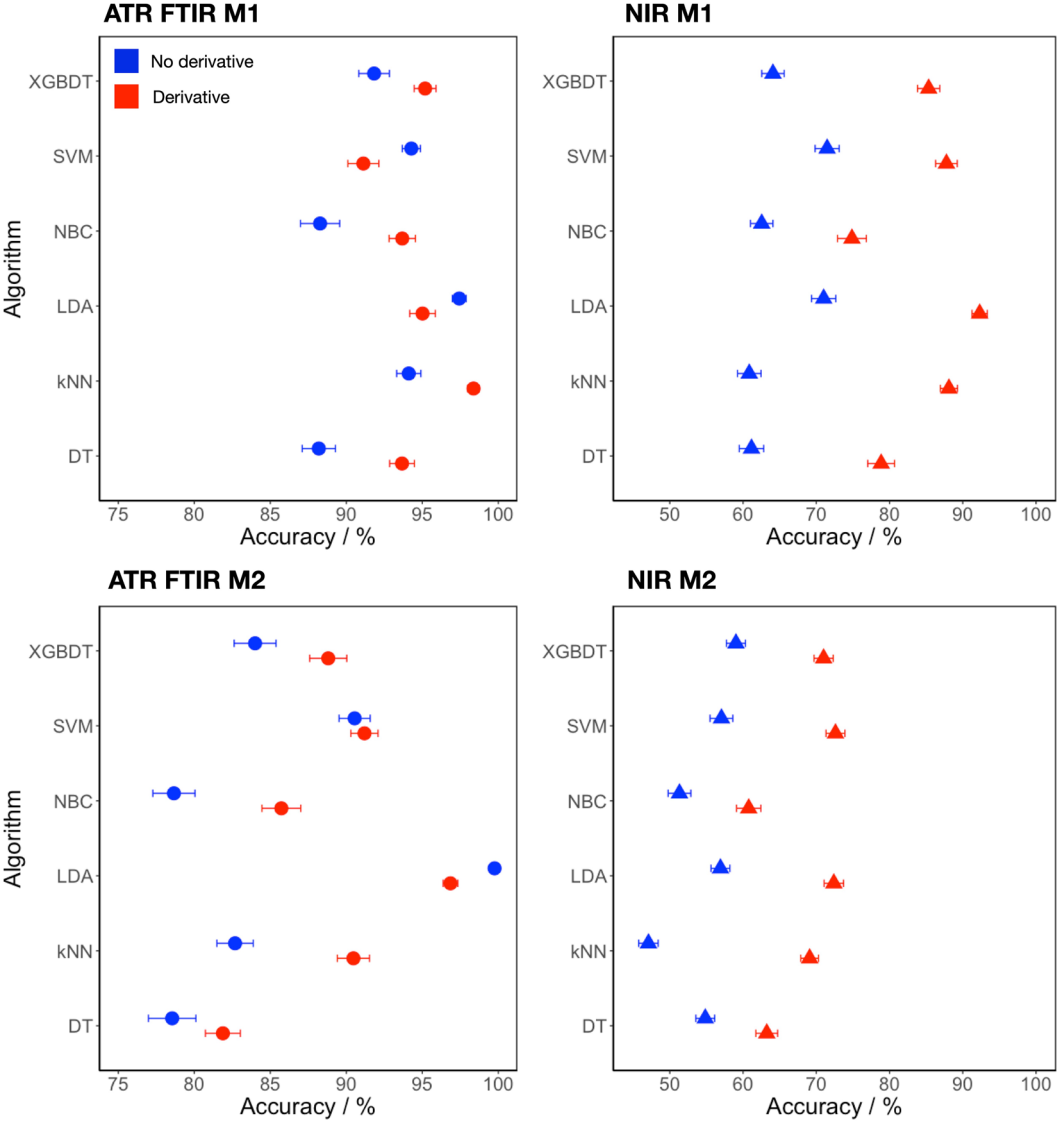
Classification accuracies of different classification algorithms used in the benchmarking procedure for the M1 and M2 classification models. Left: ATR FTIR models, right: NIR models. Blue is used to depict spectra, not processed with numerical differentiation, while red presents numerically differentiated spectra. The width of the interval presents the 95% confidence interval with the average at the centre.

The dataset generated by chromatographic and spectroscopic analysis was used as input for classification models (Supplementary Table S3). The spectral data was subjected to a dimensionality reduction according to Supplementary Table S4. The workflow is presented in Supplementary Fig. S5. Two main classification models were created (Fig. 3 and Supplementary Table S2). An object was considered to reliably contain a plasticizer at > 3% (w%). Six different classification algorithms (LDA, kNN, NBC, SVM, DT and XGBDT) were compared for classification. Some algorithms require tuneable hyperparameters that must be determined beforehand. Their values and the determination procedure are described in detail in Supplementary Table S5. The classification accuracies of M1 and M2 models are presented in Fig. 4. The exact values can be found in Supplementary Tables S6 and S7.

In general, higher classification accuracy was observed when using ATR FTIR spectra than with NIR spectra. Numerical differentiation significantly improved classification with NIR spectra, while differentiation with ATR FTIR spectra had a limited effect. The kNN algorithm produced the best model for ATR FTIR M1 spectra with numerical differentiation, according to the mean classification accuracy with a 95% confidence interval, (Fig. 4). The ANOVA F-test is significant (*p* < 2E−16), confirming that the six algorithms do not produce equally effective models. The pairwise differences between the mean accuracies of the kNN algorithm and the other five algorithms show that the results produced by kNN are significantly different (*p* >>  0.05), indicating that kNN is the most suitable algorithm (Fig. S6). Similarly, we can compare the accuracies for ATR FTIR M1 spectra without numerical differentiation. Here, the best model is obtained with LDA. The comparison of the accuracies obtained with kNN (der.) and LDA (no der.) shows that the algorithms give significantly different results (*p* = 0.002), indicating that the kNN algorithm (der.) gives the best model for ATR FTIR M1 spectra with an accuracy of 98.4%. However, it should be noted that the model using the LDA algorithm also yields a very good model with 97.4% accuracy. The same analysis can be performed for ATR FTIR M2 spectra. The LDA algorithm gives the best results with or without numerical differentiation, with the best result obtained without numerical differentiation (99.8%). As mentioned earlier, NIR spectra with numerical differentiation produce models with significantly higher classification accuracy. The LDA algorithm produces the best model when NIR M1 spectra with numerical differentiation are used (92.3%). When NIR M1 spectra without numerical differentiation are used, LDA and SVM generate models with similar (71.0% and 71.5%, respectively) but significantly worse accuracies compared to the previously mentioned model with LDA and numerical differentiation. For the NIR M2 spectra, the accuracies of the models are significantly worse than for the NIR M1 spectra. Regardless of numerical differentiation, the best models are created using the LDA, XGBDT, and SVM algorithms. The use of derivative spectra leads to significantly higher accuracies (72.3%, 70.5%, and 72.6%, respectively) compared to the use non derivative spectra (56.9%, 59.0%, and 57.1%, respectively).

**Figure 5:**
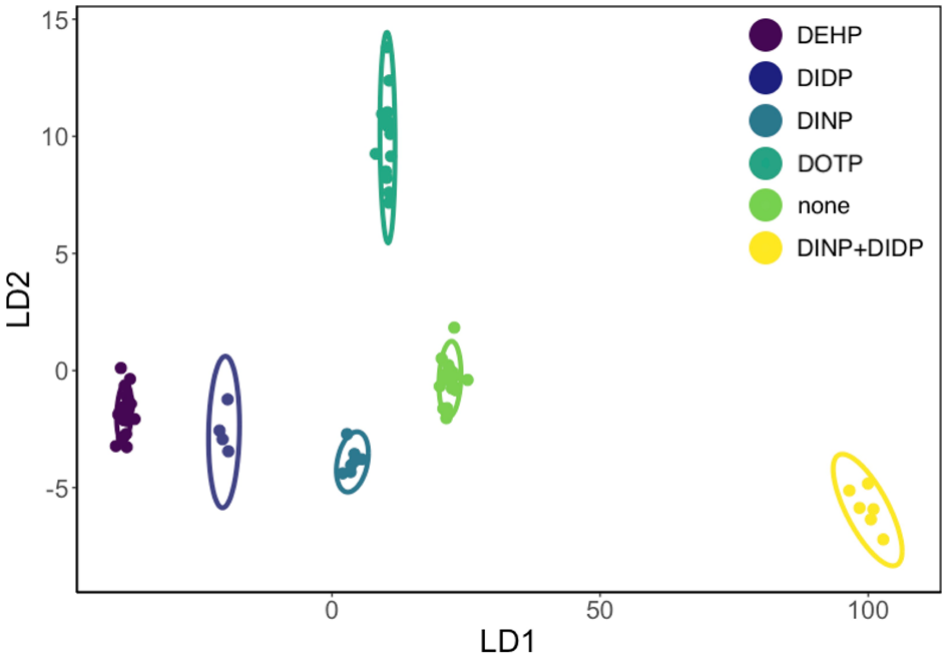
Visualisation of the most accurate LDA classification algorithm of the M2 classification model with ATR FTIR spectra, untreated with numerical differentiation (LDA ATR FTIR model M2).

Based on the comparison above, we can conclude that class prediction for real objects with single (DEHP or DOTP) or no added plasticizer in models M1 is very good using either ATR FTIR or numerically differentiated NIR spectra. Analysis of the relative confusion matrices for best models for M1 models, presented in Supplementary Table S8 confirms that all groups (DEHP, DOTP and none) have similar prediction errors. Adding objects with DINP and DOTP, as a single or as a mix, decreased the classification accuracies for both ATR FTIR and NIR spectra. As mentioned earlier, an exception to this trend was the LDA ATR FTIR model M2 (no der.), which achieved an almost perfect classification accuracy of 99.8%. Figure 5 represents a projection of the data onto the first two discriminant axes with 95% confidence ellipses around each group. All six groups are tight and clearly separated from each other. Moreover, the mixed group (DINP and DIDP) is the most dissimilar to the groups with only one plasticizer. The classification accuracies of models M2 using numerically differentiated NIR spectra and the LDA, XGBDT, and SVM algorithms are significantly worse than with the ATR FTIR LDA model. More importantly, the model misclassifies DIDP, DINP and the mixed DIDP + DINP to a notable extent, e.g., with LDA DIDP, DINP and DIDP + DINP are predicted with only 14.2%, 15.3%, and 13.3% accuracy, respectively (Supplementary Table S9). This data suggests that the NIR spectra of this dataset cannot be used to efficiently separate single plasticizers from mixtures.

Subsequent mathematical analysis of PCA projections for DEHP containing PVC objects (Supplementary Figs. S7 and S8) investigated DEHP absorption bands for the purpose of unsupervised ATR FTIR and NIR spectral band assignation. This procedure confirmed characteristic DEHP absorptions within ATR FTIR spectra and identified a band of principal DEHP absorption in NIR spectra.

### PLS regression: plasticizer quantification

**Figure 6:**
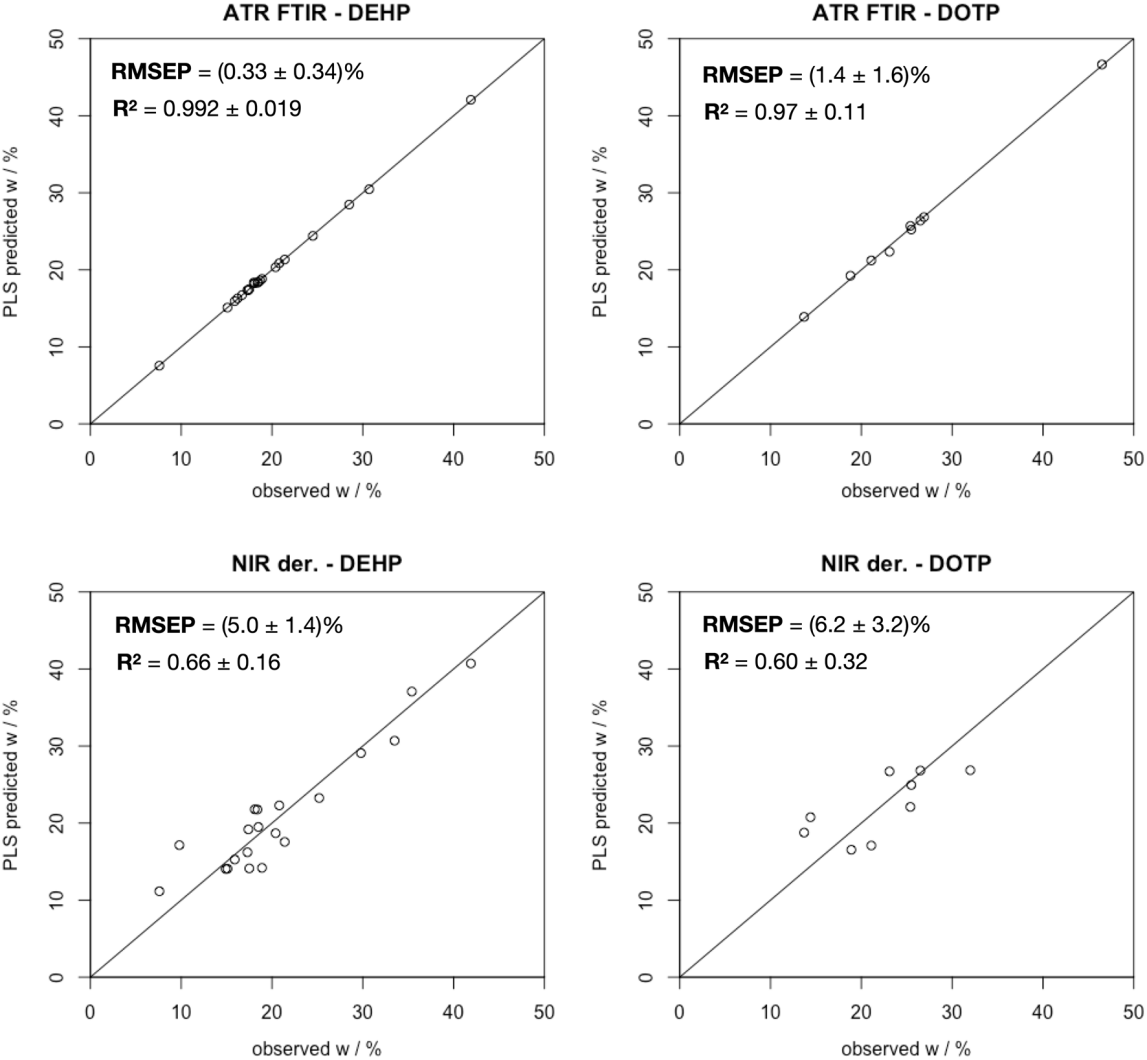
Predicted versus observed w% of DEHP and DOTP for ATR FTIR and numerically differentiated NIR models. Greater linearity indicated by higher R^2^ and lower RMSEP values indicates a greater predictive power using ATR FTIR spectra.

Two PLS regression models were created for the quantitative determination of DEHP and DOTP in PVC objects based only on their NIR and ATR FTIR spectra. The effect of numerical differentiation of the spectra on the root mean squared error of prediction (RMSEP) and correlation coefficient (R^2^) metrics were also investigated (Fig. 6). All of the resulting metrics for all the different PLS regression models under investigation are presented in Supplementary Table S10, Figs. S9 and S10. A narrower RMSEP interval was observed for DEHP than for DOTP (Supplementary Figs. S9 and S10), probably because the number of objects containing DEHP is twice that of objects containing DOTP. Higher R^2^ values and lower RMSEP values for ATR FTIR spectra are to be expected because the spectra contain a larger number of absorption frequencies of different chemical species and thus more information about their quantity^53^. Figure 6 further illustrates this trend as better linearity is observed for ATR FTIR models, which contrasts with more scatter in the NIR der. models. Using derivative ATR FTIR spectra does not improve the model, whereas an improvement in model performance is observed for numerically differentiated NIR spectra. Although numerically differentiated NIR spectra yield poorer model performance than ATR FTIR, their RMSEP values suggest that model uncertainty is in the range of expected experimental uncertainty, i.e., (5 ± 1)% and (6 ± 3)% for DEHP and DOTP regression model, respectively.

## Conclusions

Destructive and non-destructive methods of chemical analysis with machine learning approaches using spectroscopic data were used to obtain information previously available only in a destructive manner. Previous studies using a limited number of custom-made samples containing single analytes were considered inadequate for analysing diverse, real PVC objects. Therefore, we built a large collection of more than 100 PVC objects differing in their degradation stages, histories of storage, chemical composition, and thickness. The objects were destructively analysed with gas chromatography to obtain information on the identity and content of plasticizers. This information was used to create a publicly available dataset, to be expanded as the collection continues to grow. The currently available data was used together with the ATR FTIR and NIR spectra to create classification and regression models for the most common plasticizers.

In this study, we presented a classification model to identify DEHP, DOTP, DINP, DIDP, a mixture of DINP with DIDP, and unplasticized PVC. The model is capable of identifying separate plasticizers apart from a mixture of two plasticizers. Only the combination of DINP and DIDP was investigated because objects containing other combinations of plasticizers were too rare. Successful regression models were built for DEHP and DOTP, the most common plasticizers found in our collection of modern and historical PVC objects.

Numerical differentiation proved to be particularly useful for NIR spectra, as it increased the classification accuracy. Both types of spectra can be used for spectral quantification using PLS regression, but NIR spectra result in less favourable RMSEP values. Overall, the machine learning classification and regression models built with ATR FTIR spectra are more accurate and more robust than with NIR spectra. We hypothesize that the reason is that ATR FTIR spectra contain more and better-resolved information about the chemical composition and molecular structure of the object compared to NIR spectra. This study demonstrates that robust classification and regression models can be built based on collections of varied real-life objects.

Recent research on long-term PVC degradation suggests that knowing the identity of a plasticizer is important in studies of plasticizer loss and associated conservation challenges. Knowing the plasticizer also helps as a rough estimate of age: In the EU, objects with DBP and DEHP tended to be manufactured before 2008, while those with DOTP, DINP, DIDP, and DINCH mostly after 2008.

## Materials and methods

### Materials and chemicals

Commercial diisononyl phthalate (DINP), di(2-propylheptyl) phthalate (DPHP), and diisononyl cyclohexane-1,2-dicarboxylate (DINCH) by BASF were kindly supplied by OQEMA. Dibutyl phthalate (DBP, 99.5%), di(2-ethylhexyl) phthalate (DEHP, ≥ 99.5) and dioctyl terephthalate (DOTP, ≥ 96%) were purchased from Sigma-Aldrich (Germany). Diisodecyl phthalate (DIDP) was purchased from TCI (Japan). Inhibitor-free tetrahydrofurane (THF, Sigma-Aldrich, > 99.9%) hexane (Honeywell, > 95%) and acetone (Honeywell, ≥ 99.8%) were used. 22 mm 0.45 μm nylon filters and 2 mL DEHP-free and PVC-free syringes (Chirana T. Injecta) were used for filtration.

1 mg/mL stock solutions of DBP, DEHP, DOTP, DPHP, DINP, DIDP, and DINCH were prepared in acetone. Working solution for calibration were prepared daily by dilution in hexane for GC-FID and GC–MS analysis.

### Collection of PVC objects

A collection of 103 PVC objects with different plasticizers, their content, transparent or coloured, new or historical (presented in more details in further section and Supplementary Tables S1 and S2) was characterised destructively with gas chromatography with two different detectors (FID and MS) and non-destructively with NIR and ATR-IR spectroscopy. The collection of PVC objects, ranging from new to 30 years old, was gathered by donation and is available for further research.

### FTIR analysis

FTIR spectra were recorded using a Perkin Elmer Spectrum Two FT-IR Spectrometer with an attenuated total reflectance accessory (ATR). The spectra were recorded between 4000 and 450 cm^−1^ with a 4 cm^− 1^ spectral resolution. For each spectrum, 10 scans were co-added. As a background spectrum, air was used. Samples with appropriate geometry were analysed by clamping them into the ATR accessory without further preparation, while bulky objects had a suitably small piece cut off. The surface of objects with visible dirt depositions was removed.

### NIR analysis

NIR spectra were recorded using a portable spectrometer ASD LabSpec 5000 with a built-in light source and fibreoptic cables. Spectra were acquired in a wavelength range of 350–2500 nm with a sampling interval of 1 nm. For the baseline, a white reference standard (Spectralon) with > 95% reflectance across the entire wavelength range was used. The spectra were recorded perpendicular to the object’s surface with the white reference standard as background. For each spectrum, 100 scans were averaged and splice correction for the light source was used to achieve a continuous spectrum.

### Sample preparation

A small amount (10 mg) of PVC sample was accurately weighed into a glass vial and dissolved in 1 mL of THF by shaking for 1 h. Some solutions were transparent, while others were opaque due to the presence of insoluble additives. 2 mL of hexane were added to precipitate polyvinyl chloride. The suspension was filtered through a 0.45 μm nylon filter and diluted with hexane for GC-FID or GC–MS analysis.

### Chromatographic determination of plasticizer identity and content

GC-FID analyses were performed with a Trace 1300 Gas Chromatograph (Thermo Fisher Scientific, USA), with a flame-ionization detector (FID). Helium was used as a carrier gas (constant flow 2.5 mL/min). The detector temperature was set at 320 °C. The injection was performed with a split ratio of 25 at 320 °C. The injected volume was 1 μL. Chromatographic separations were performed on a Restek Rxi-5Sil MS capillary column (30 m, 0.32 mm i.d., 0.25 μm film thickness). The temperature was set as 100 °C for 1 min, 40 °C/min until 320 °C and maintaining the final temperature for 2.5 min. Retention times are presented in Supplementary Fig. S2.

GC–MS analyses were performed on a Thermo Scientific™ TSQ™ 9000 triple quadrupole GC–MS/MS (Thermo Fisher Scientific, USA) in positive ion mode with electron ionization energy 70 eV. Some parameters of the GC-FID method were adapted due to using MS as a detector. Carrier gas constant flow was decreased to 1.2 mL/min. The transfer line was held at 320 °C. The ion source temperature was set at 250 °C. Chromatographic separations were performed on an Agilent capillary column HP-5MS (30 m, 0.25 mm i.d., 0.25 μm film thickness). The temperature gradient was modified due to decreased flow of carrier gas: 100 °C for 1 min, 30 °C/min until 320 °C and maintaining the final temperature for a total run-time of 13 min. The solvent delay was 2.5 min. Screening of plasticizers in PVC extracts was performed as a full scan (*m/z* 40–350). Quantification of plasticizer was performed using SIM with a dwell time of 50 ms for each ion.

### Multivariate analysis and machine learning

Multivariate analysis and machine learning approaches were implemented using R and are described in detail in the Supplementary Information Section 4 and Supplementary Tables S3 and S4. A spectral pre-treatment workflow was developed to create spectra that can be readily used in subsequent machine learning algorithms. Two supervised classification models were created, utilizing four different types of spectra: ATR FTIR and NIR spectra with and without numerical derivation as a pre-processing step. Two PLS regression models were created to quantify the two most predominant plasticizers in PVC objects, utilizing the same four different types of spectra.

## Supplementary Information

Supplementary Information 1.

Supplementary Information 2.

## Data Availability

The data analysed during the current study is available from the Repository of the University of Ljubljana (https://repozitorij.uni-lj.si/IzpisGradiva.php?id=134404).
