# Supplementary material for: Machine learning-assisted non-destructive plasticizer identification and quantification in historical PVC objects based on IR spectroscopy
Source: Sci Rep. 2022 Mar 23;12:5017. doi: 10.1038/s41598-022-08862-1 (PMC8943100; doi:10.1038/s41598-022-08862-1)
Supplement: Supplementary file 2 — Supplementary Information 2. [file 41598_2022_8862_MOESM2_ESM.pdf]

Supplementary information for:

Machine learning-assisted non-destructive plasticizer identification and quantification in historical PVC objects based on IR spectroscopy

Tjaša Rijavec[1,*], David Ribar[1], Jernej Markelj[1], Matija Strlič[1,2,3], Irena Kralj Cigić[1]

[1]*Faculty of Chemistry and Chemical Technology, University of Ljubljana, Ljubljana, Slovenia*
[2]*Institute for Sustainable Heritage, University College London, London, UK*
[3]*Museum Conservation Institute, Smithsonian Institution, Suitland MD, USA*

*Correspondence: tjasa.rijavec@fkkt.uni-lj.si

Table of Contents

1. Investigated plasticizers 2

2. Identification and quantification of plasticizers by gas chromatography 3

3. Description of the PVC samples 4

4. Multivariate analysis and machine learning 8

4.1. Used software and packages 8

4.2. Information on input dataset used in MVA and ML 8

4.3. Workflow presentation 8

4.4. Spectral pre-treatment 10

4.5. Supervised classification algorithm 10

4.6. Regression algorithm 10

5. Additional outcomes of statistical evaluation 13

5.1. Classification accuracies 13

5.2. Comparison of classification models 13

5.3. Confusion matrices 15

5.5. Regression results 19

1. Investigated plasticizers

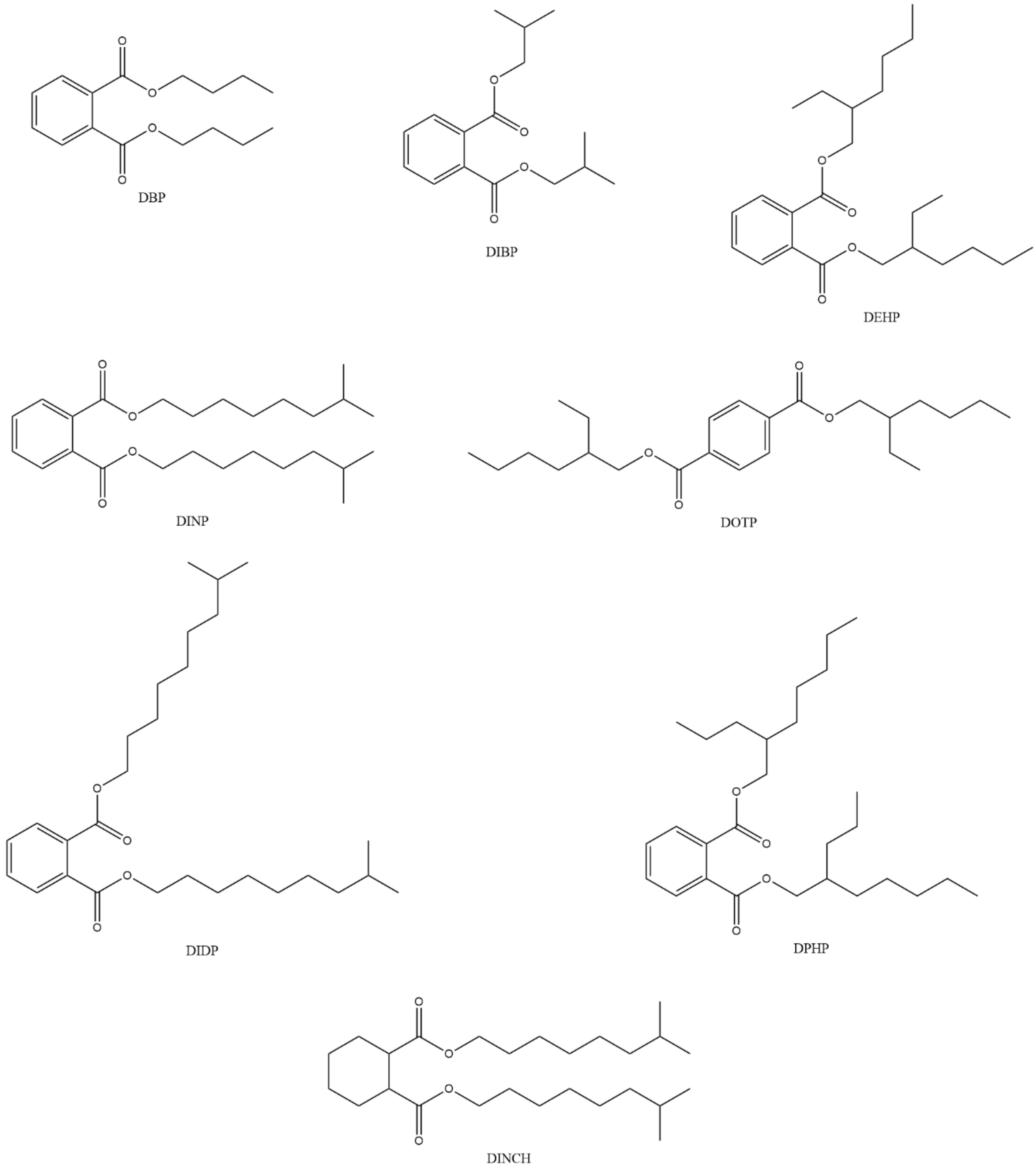

Figure S1: Chemical structures of the investigated plasticizers.

2. Identification and quantification of plasticizers by gas chromatography

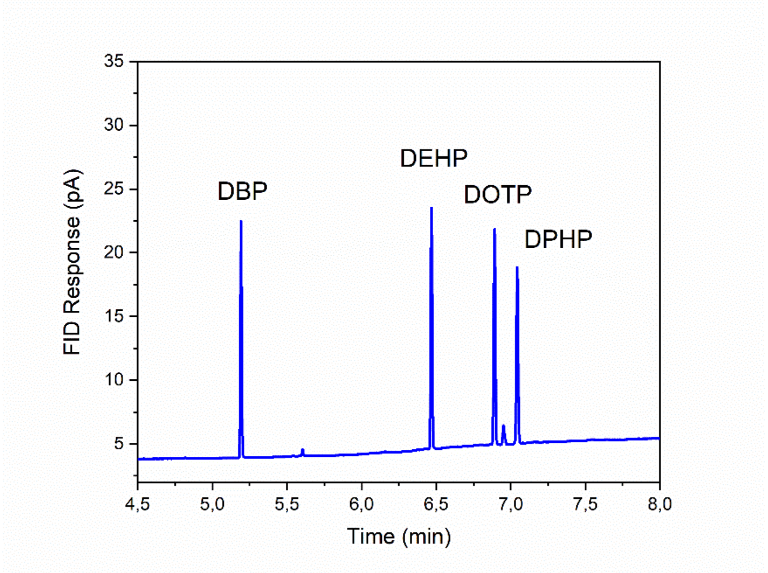

Figure S2: GC-FID chromatogram after injection of a standard solution with 30 mg/L of each individual plasticizer.

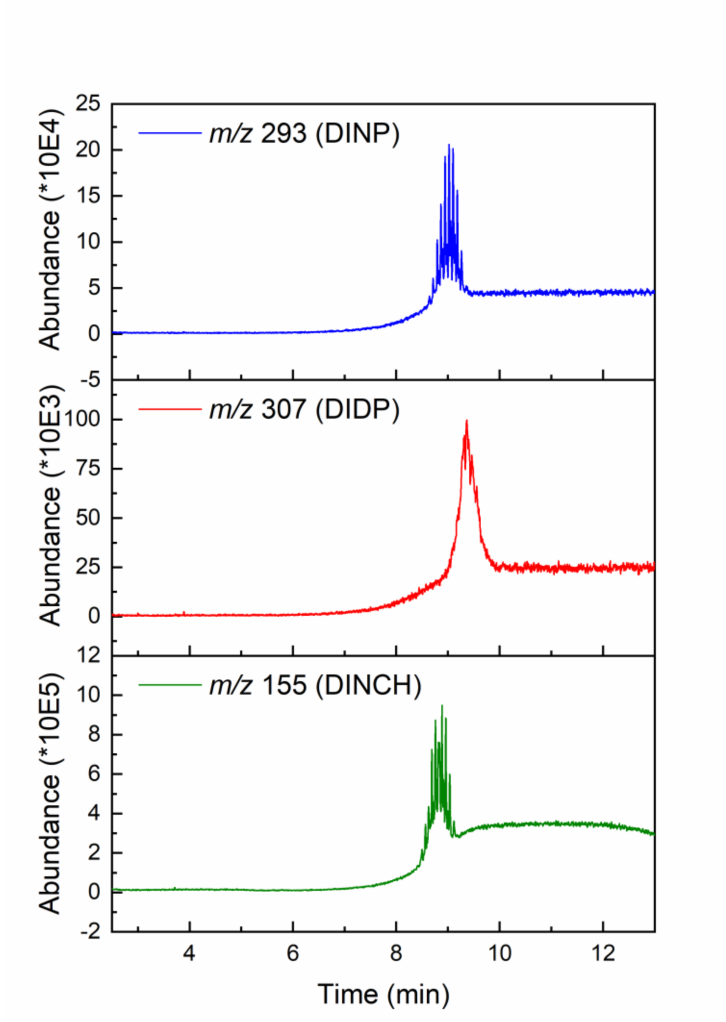

Figure S3: SIM chromatograms after injection of standard solutions with 5 mg/L of individual plasticizers.

3. Description of the PVC samples

Table S1: The PVC sample set with the identified plasticizers and their respected content (in w%). The column C/L shows which objects were coloured (C = 1) or colourless (L=0). The column H/M shows which objects were degraded and historical (H=1) and which were contemporary (M=0). The abbreviations P1, P2 and P3 denote three identified plasticizers (where found).

ID	*All plasticizers*	*C/L*	*H/M*	*P1 ID*	*P1 (%)*	*P2 ID*	*P2 (%)*	*P3 ID*	*P3 (%)*
**1**	none	1	1	none	/	/	/	/	/
**2**	none	1	1	none	/	/	/	/	/
**3**	none	0	1	none	/	/	/	/	/
**4**	none	0	1	none	/	/	/	/	/
**5**	none	1	1	none	/	/	/	/	/
**6**	none	0	1	none	/	/	/	/	/
**7**	none	0	1	none	/	/	/	/	/
**8**	none	0	1	none	/	/	/	/	/
**9**	none	0	1	none	/	/	/	/	/
**10**	none	0	1	none	/	/	/	/	/
**11**	none	0	1	none	/	/	/	/	/
**12**	none	0	1	none	/	/	/	/	/
**13**	none	0	1	none	/	/	/	/	/
**14**	none	0	1	none	/	/	/	/	/
**15**	none	0	1	none	/	/	/	/	/
**16**	none	0	1	none	/	/	/	/	/
**17**	none	0	1	none	/	/	/	/	/
**17**	none	0	1	none	/	/	/	/	/
**19**	none	0	1	none	/	/	/	/	/
**20**	none	0	1	none	/	/	/	/	/
**21**	none	0	1	none	/	/	/	/	/
**22**	none	1	1	none	/	/	/	/	/
**23**	none	1	1	none	/	/	/	/	/
**24**	none	1	1	none	/	/	/	/	/
**25**	none	0	0	none	/	/	/	/	/
**26**	DEHP	0	1	DEHP	15.9	/	/	/	/
**27**	DEHP	0	1	DEHP	17.4	/	/	/	/
**28**	DEHP	0	1	DEHP	17.3	/	/	/	/
**29**	DEHP	0	1	DEHP	16.4	/	/	/	/
**30**	DEHP	0	1	DEHP	20.4	/	/	/	/
**31**	DEHP	1	1	DEHP	20.8	/	/	/	/
**32**	DEHP	1	1	DEHP	35.4	/	/	/	/
**33**	DEHP	0	1	DEHP	25.2	/	/	/	/
**34**	DEHP	0	1	DEHP	7.6	/	/	/	/
**35**	DEHP	0	1	DEHP	18.7	/	/	/	/
**36**	DEHP	0	1	DEHP	17.5	/	/	/	/
**37**	DEHP	0	1	DEHP	9.8	/	/	/	/
**38**	DEHP	1	1	DEHP	33.7	/	/	/	/
**39**	DEHP	0	1	DEHP	20.8	/	/	/	/

**40**	DEHP	0	1	DEHP	18.1	/	/	/	/
**41**	DEHP	0	1	DEHP	18.4	/	/	/	/
**42**	DEHP	0	1	DEHP	16.2	/	/	/	/
**43**	DEHP	0	1	DEHP	18.5	/	/	/	/
**44**	DEHP	0	1	DEHP	19.3	/	/	/	/
**45**	DEHP	1	1	DEHP	41.9	/	/	/	/
**46**	DEHP	1	1	DEHP	24.5	/	/	/	/
**47**	DEHP	0	1	DEHP	18.0	/	/	/	/
**48**	DEHP	1	1	DEHP	21.4	/	/	/	/
**49**	DEHP	0	1	DEHP	18.9	/	/	/	/
**50**	DEHP	0	1	DEHP	15.1	/	/	/	/
**51**	DEHP	0	1	DEHP	17.8	/	/	/	/
**52**	DEHP	0	1	DEHP	14.9	/	/	/	/
**53**	DEHP	0	1	DEHP	16.7	/	/	/	/
**54**	DEHP	1	0	DEHP	30.7	/	/	/	/
**55**	DEHP	1	0	DEHP	29.8	/	/	/	/
**56**	DEHP	1	0	DEHP	33.5	/	/	/	/
**57**	DEHP	1	0	DEHP	28.5	/	/	/	/
**58**	DOTP	1	1	DOTP	12.3	/	/	/	/
**59**	DOTP	0	0	DOTP	18.9	/	/	/	/
**60**	DOTP	0	0	DOTP	21.1	/	/	/	/
**61**	DOTP	0	0	DOTP	21.1	/	/	/	/
**62**	DOTP	0	0	DOTP	26.9	/	/	/	/
**63**	DOTP	1	0	DOTP	46.5	/	/	/	/
**64**	DOTP	1	0	DOTP	14.4	/	/	/	/
**65**	DOTP	1	1	DOTP	25.4	/	/	/	/
**66**	DOTP	1	1	DOTP	23.1	/	/	/	/
**67**	DOTP	1	1	DOTP	18.8	/	/	/	/
**68**	DOTP	1	1	DOTP	25.5	/	/	/	/
**69**	DOTP	1	1	DOTP	26.5	/	/	/	/
**70**	DOTP	1	1	DOTP	32.0	/	/	/	/
**71**	DEHP	1	0	DOTP	13.7	/	/	/	/
**72**	DINP	1	1	DINP	22.2	/	/	/	/
**73**	DINP	1	1	DINP	33.7	/	/	/	/
**74**	DINP	1	1	DINP	27.0	/	/	/	/
**75**	DINP	1	1	DINP	38.1	/	/	/	/
**76**	DINP	1	1	DINP	34.3	/	/	/	/
**77**	DINP	1	1	DINP	29.5	/	/	/	/
**78**	DINP	1	1	DINP	34.6	/	/	/	/
**79**	DIDP	0	1	DIDP	13.6	/	/	/	/
**80**	DIDP	0	1	DIDP	15.4	/	/	/	/
**81**	DIDP	1	1	DIDP	23.7	/	/	/	/
**82**	DIDP	1	1	DIDP	28.9	/	/	/	/
**83**	DINCH	1	1	DINCH	30.5	/	/	/	/
**84**	DINP + DIDP	1	1	DINP	17.1	DIDP	6.7	/	/
**85**	DINP + DIDP	1	1	DINP	23.9	DIDP	9.5	/	/

**86**	DINP + DIDP	1	1	DINP	30.6	DIDP	14.0	/	/
**87**	DINP + DIDP	1	1	DINP	22.7	DIDP	7.8	/	/
**88**	DINP + DIDP	1	1	DINP	24.5	DIDP	9.9	/	/
**89**	DINP + DIDP	1	1	DINP	32.2	DIDP	13.3	/	/
**90**	DBP + DINP	1	1	DBP	7.1	DINP	16.5	/	/
**91**	DBP + DINP	1	1	DBP	6.7	DINP	34.9	/	/
**92**	DBP + DINP	1	1	DBP	2.2	DINP	15.3	/	/
**93**	DBP + DINP + DIDP	1	1	DBP	5.4	DINP	16.5	DIDP	6.7
**94**	DEHP + DINP + DIDP	1	1	DEHP	4.7	DINP	40.0	DIDP	13.5
**95**	DEHP + DINP + DIDP	1	1	DEHP	15.4	DINP	24.4	DIDP	10.0
**96**	DEHP + DINP + DIDP	1	1	DEHP	3.6	DINP	21.6	DIDP	7.0
**97**	DEHP + DINP	1	1	DEHP	2.6	DINP	33.6	/	/
**98**	DEHP + DBP	1	1	DEHP	40.2	DBP	0.3	/	/
**99**	DEHP + DBP	0	1	DEHP	16.9	DBP	0.7	/	/
**100**	DEHP + DIBP	1	1	DEHP	23.4	DIBP	10.4	/	/
**101**	DEHP + DIBP	1	1	DEHP	8.9	DIBP	1.0	/	/
**102**	DEHP + DIDP	0	1	DEHP	3.4	DIDP	14.4	/	/
**103**	DEHP + DOTP + DPHP	0	1	DOTP	14.1	DPHP	1.8	DEHP	0.6

***/ Not present or** below the limit of detection

Table S2. Summary table of the results of plasticizer identification by gas chromatography for the PVC sample set. Classification models M1 and M2 were developed for objects with the types of plasticizers as indicated.

	**Plasticizer**	**Count**	**M1**	**M2**
	None	25	y	y
**Single**	DEHP	32	y	y
	DOTP	14	y	y
	DINP	7	n	y
	DIDP	4	n	y
	DINCH	1	n	n
**Two**	DINP + DIDP	6	n	y
	DBP + DINP	3	n	n
	DEHP + DBP	2	n	n
	DEHP + DIBP	2	n	n
	DEHP + DIDP	1	n	n
	DEHP + DINP	1	n	n
**Three**	DEHP + DINP + DIDP	3	n	n
	DBP + DINP + DIDP	1	n	n
	DEHP + DOTP + DPHP	1	n	n
	Single or None =	83		
	Mix (2 or 3) =	20		
	Total =	103	71	88

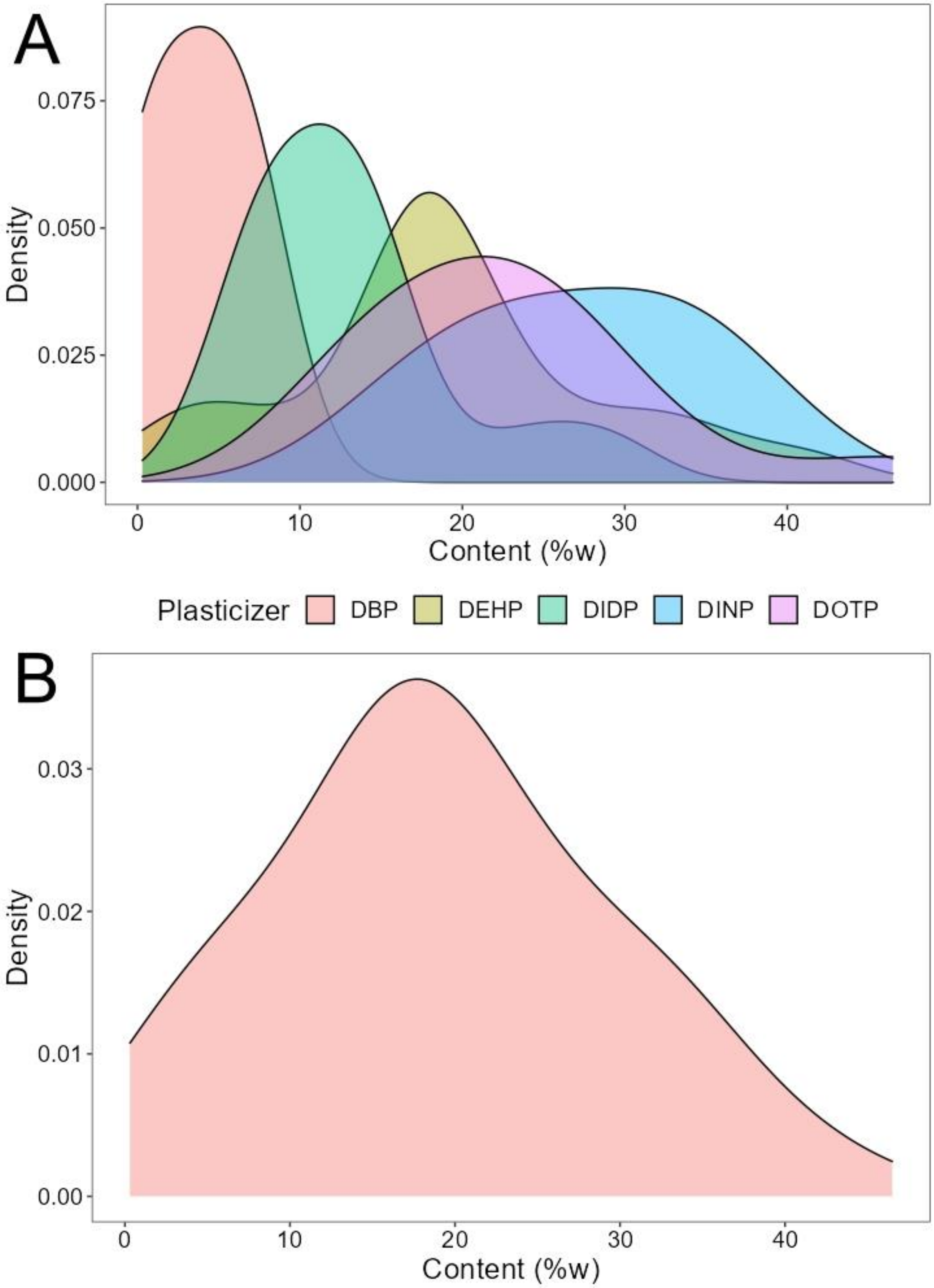

Figure S4: Density distributions (> 0.1 %w) for (A) each plasticizer separately: DBP (6), DEHP (42), DIDP (15), DINP (21), DOTP (15), and (B) for all the plasticizers. Note that objects with DIBP, DINCH and DPHP are not included in (A) since they are contained in max. 2 objects.

4. Multivariate analysis and machine learning

4.1. Used software and packages

Multivariate analysis and machine learning was implemented using R[1] (ver. 4.0.5) in Rstudio[2] (1.4.1106). The algorithm also includes these libraries:

- tidyverse[3] (ver. 1.3.1) for pipeline design
- mlr[4] (ver. 2.19.0) for machine learning
- prospectr[5] (ver. 0.2.1) for digital filters
- pls[6] (ver. 2.7.3) for regression analysis
- caTools[7] (ver. 1.18.2) for regression analysis
- viridis[8] (ver. 0.6.1) for graphics production
- ggplot2[9] (ver. 3.3.3) for graphics production

4.2. Information on input dataset used in MVA and ML

Table S3: Dataset type information for all input parameters.

Input/Output parameter	Method of acquisition	Dataset Type
Plasticizer Content	GC-FID or GC-MS	Numeric
FTIR Spectra	ATR-FTIR Spectrometer	Continuous
NIR Spectra	NIR Spectrometer	Continuous
Plasticizer Identification	GC-MS	Categorical

4.3. Workflow presentation

[1] R Core Team (2021). "R: A language and environment for statistical computing." R Foundation for Statistical Computing, Vienna, Austria. URL. https://www.R-project.org/

[2] RStudio Team (2021). "RStudio: Integrated Development Environment for R. RStudio", PBC, Boston, MA. http://www.rstudio.com/

[3] Wickham H, Averick M, Bryan J, Chang W, McGowan LD, François R, Grolemund G, Hayes A, Henry L, Hester J, Kuhn M, Pedersen TL, Miller E, Bache SM, Müller K, Ooms J, Robinson D, Seidel DP, Spinu V, Takahashi K, Vaughan D, Wilke C, Woo K, Yutani H (2019). "Welcome to the tidyverse." Journal of Open Source Software, 4(43), 1686. doi: 10.21105/joss.01686

[4] Bischl B, Lang M, Kotthoff L, Schiffner J, Richter J, Studerus E, Casalicchio G, Jones Z (2016). "mlr: Machine Learning in R." Journal of Machine Learning Research, 17(170), 1-5. https://jmlr.org/papers/v17/15-066.html

[5] Stevens A, Ramirez-Lopez L (2021). "An introduction to the prospectr package". https://cran.r-project.org/web/packages/prospectr/vignettes/prospectr.html

[6] Hovde Liland K, Mevik B-H, Wehrens R, Hiemstra P (2021). "pls: Partial Least Squares and Principal Component Regression". https://CRAN.R-project.org/package=pls

[7] Tuszynski J (2021). "caTools: Tools: Moving Window Statistics, GIF, Base64, ROC AUC, etc". https://CRAN.R-project.org/package=caTools

[8] Garnier S, Ross N, Rudis R, Camargo P A, Sciaini M, Scherer C (2021). "viridis - Colorblind-Friendly Color Maps for R". doi: 10.5281/zenodo.4679424, https://sjmgarnier.github.io/viridis/

[9] Wickham H (2016). "ggplot2: Elegant Graphics for Data Analysis". Springer-Verlag New York. ISBN 978-3-319-24277-4. https://ggplot2.tidyverse.org

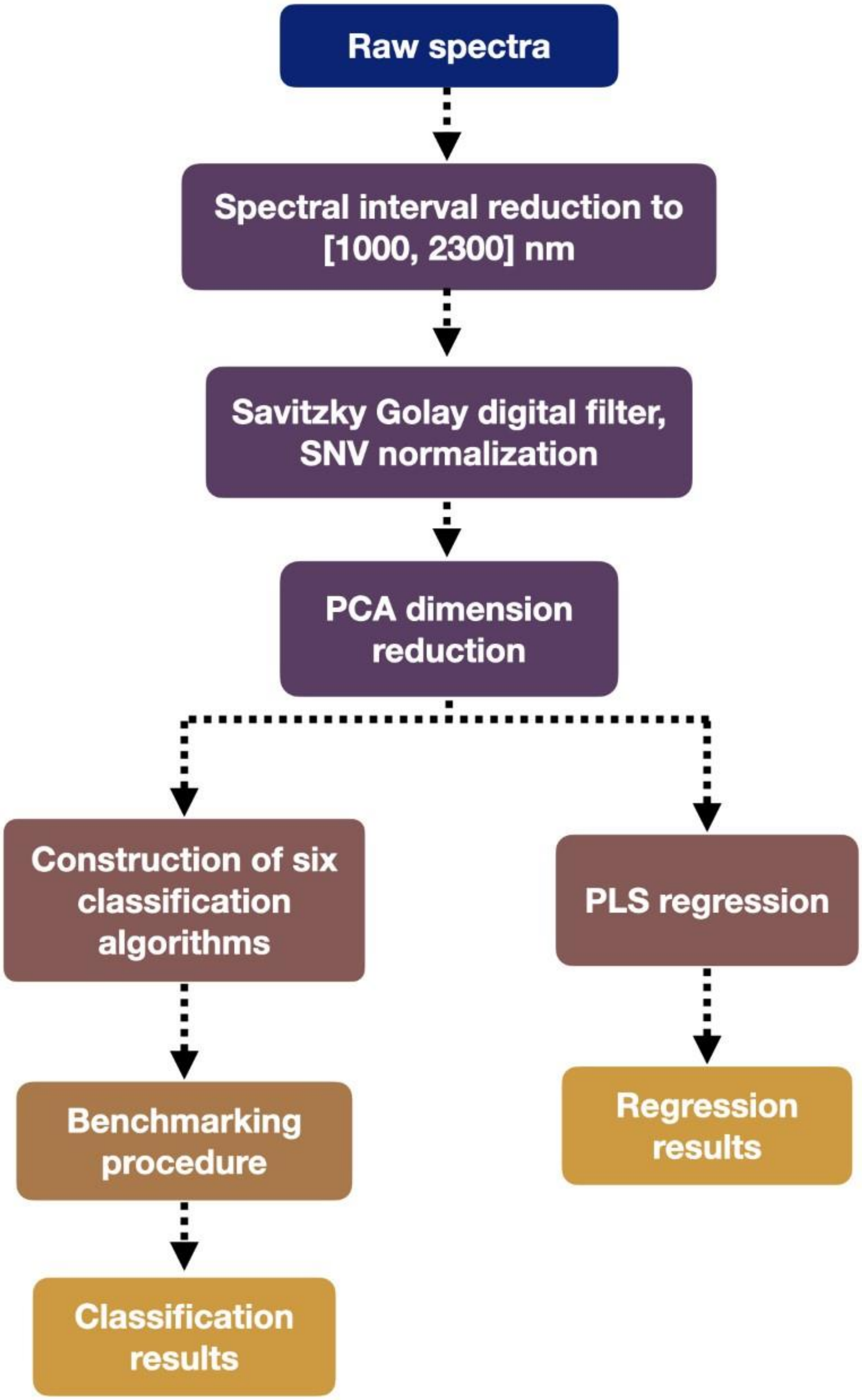

Figure S5: Pipeline diagram presenting the workflow of building classification and regression models.

4.4. Spectral pre-treatment

Each experimental NIR spectrum was narrowed to the spectral interval of [1000, 2300] nm and passed through a Savitzky Golay digital filter utilizing a second-degree polynomial at a width interval equalling 0.85% of the total interval. Derivatives of the spectra were also created using a modified SG filter.

The spectra were normalised using a standard normal variate filter, to create comparable datasets. Principal component analysis was used for dimensionality reduction of the dataset. The number of principal components selected varied amongst different types of spectra, as only the principal components, of which eigenvalue exceeded 1, were used.

In Supplementary Table S4, the numbers of selected principal components are listed.

Table S4: Selected principal components for each model and type of spectra.

	**DEHP regression model**	**DOTP regression model**	**Classification model M1**	**Classification model M2**
**ATR FTIR**	8	10	11	12
**ATR FTIR + der.**	11	9	16	18
**NIR**	5	4	6	6
**NIR + der.**	10	9	13	14

4.5. Supervised classification algorithm

After the PCA dimensionality reduction, each spectrum was labelled according to the type of plasticizer. Six different classification algorithms were utilized:

- k-nearest neighbours (kNN)
- support vector machines (SVM)
- linear discriminant analysis (LDA)
- naïve Bayes classification (NBC)
- decision trees (DT)
- extreme gradient boosted decision trees (XGBDT)

For the first model M1, 71 spectra were used. For the second model M2, 88 spectra were used.

The kNN, SVM, DT and XBG, which all require additional hyperparameters, were optimised using a 100-iteration random Bootstrap method. The optimised hyperparameters can be seen in Supplementary Table S4.

The final benchmarking procedure was implemented using the Bootstrap method of 100 iterations and stratification, to ensure the relative percentage of plasticizer type remains constant. During each iteration, all six classification algorithms were assessed in the accuracy of their prediction. The result is an average of all iterations, along with the standard deviation of 100 iterations.

4.6. Regression algorithm

A custom regression algorithm was developed. After the PCA dimensionality reduction, only spectra containing DEHP and DOTP were selected, each for one regression model.

The spectra of 32 DEHP and 14 DOTP containing samples were used.

A 100-iteration loop was used to construct 100 different partial least squares regression models, with a 70/30 partition strategy. In each iteration, 70% of randomly selected spectra were used to construct a model and the remaining 30% were used to test the root mean squared error of prediction. The result is an average of all iterations, along with the corresponding standard deviations.

Table S5: Optimised hyperparameters for classification algorithms.

Hyperparameter	Classification model 1 (M1) ATR FTIR	ATR FTIR + der.	NIR	NIR + der.	Classification model 2 (M2) ATR FTIR	ATR FTIR + der.	NIR	NIR + der.	Search interval
					**kNN**				
k	1	8	1	1	1	1	2	1	1 ... 10
					**SVM**				
kernel	polynomial	polynomial	polynomial	polynomial	polynomial	polynomial	sigmoid	polynomial	polynomial, radial, sigmoid
degree	1.00	1.00	1.00	1.00	1.00	1.00	3.00	3.00	1 ... 3
cost	5.19	0.38	8.09	2.67	7.64	4.11	0.15	1.05	0.1 ... 10
gamma	4.55	3.01	3.92	0.91	2.20	0.44	0.19	2.31	0.1 ... 10
					**DT**				
minsplit	19	20	11	10	6	6	14	5	5 ... 20
minbucket	5	7	3	4	3	4	8	3	3 ... 10
cp	0.0492	0.0158	0.041	0.0338	0.0464	0.0264	0.0966	0.0951	0.01 ... 0.1
maxdepth	6	10	6	6	8	10	3	6	3 ... 10
					**XGBDT**				
eta	0.413	0.0896	0.295	0.478	0.559	0.413	0.49	0.788	0 ... 1
gamma	1.21	0.21	0.304	0.138	0.132	0.0315	2.65	0.394	0 ... 5
max_depth	1	2	2	3	2	4	4	1	1 ... 5
min_child_weight	2.6	2.32	1.68	1.99	1.99	3.12	2.74	3.69	1 ... 10
subsample	0.876	0.698	0.99	0.677	0.761	0.895	0.88	0.785	0.5 ... 1
colsample_bytree	0.687	0.902	0.774	0.584	0.729	0.921	0.935	0.619	0.5 ... 1
eval_metric	mlogloss	merror	merror	merror	mlogloss	merror	merror	merror	merror, mlogloss

5. Additional outcomes of statistical evaluation

5.1. Classification accuracies

Supplementary Tables S6 and S7 present the detailed classification accuracies along with the 95% confidence interval for both classification models.

Table S6: Results for the M1 classification model.

	Classification accuracy / %			
**Algorithm**	**ATR FTIR - No der.**	**ATR FTIR - der.**	**NIR - No der.**	**NIR - der.**
kNN	94.1 ± 0.8	98.4 ± 0.4	60.9 ± 1.6	88.1 ± 1.2
SVM	94.3 ± 0.6	91.1 ± 1.0	71.5 ± 1.6	87.8 ± 1.5
LDA	97.4 ± 0.5	95.0 ± 0.8	71.0 ± 1.7	92.3 ± 1.0
NBC	88.3 ± 1.3	93.7 ± 0.9	62.6 ± 1.5	74.9 ± 2.0
DT	88.2 ± 1.1	93.7 ± 0.8	61.2 ± 1.7	78.9 ± 1.8
XGBDT	91.8 ± 1.0	95.2 ± 0.7	64.1 ± 1.5	85.3 ± 1.5

Table S7: Results for the M2 classification model.

	Classification accuracy / %			
**Algorithm**	**ATR FTIR - No der.**	**ATR FTIR - der.**	**NIR - No der.**	**NIR - der.**
kNN	82.7 ± 1.2	90.5 ± 1.1	47.1 ± 1.3	69.1 ± 1.2
SVM	90.5 ± 1	91.2 ± 0.9	57.1 ± 1.6	72.6 ± 1.3
LDA	99.8 ± 0.2	96.9 ± 0.5	56.9 ± 1.3	72.4 ± 1.3
NBC	78.7 ± 1.4	85.7 ± 1.3	51.3 ± 1.5	60.8 ± 1.7
DT	78.5 ± 1.6	81.9 ± 1.1	54.8 ± 1.3	63.2 ± 1.5
XGBDT	83.5 ± 1.4	88.8 ± 1.2	59.0 ± 1.3	70.5 ± 1.3

5.2. Comparison of classification models

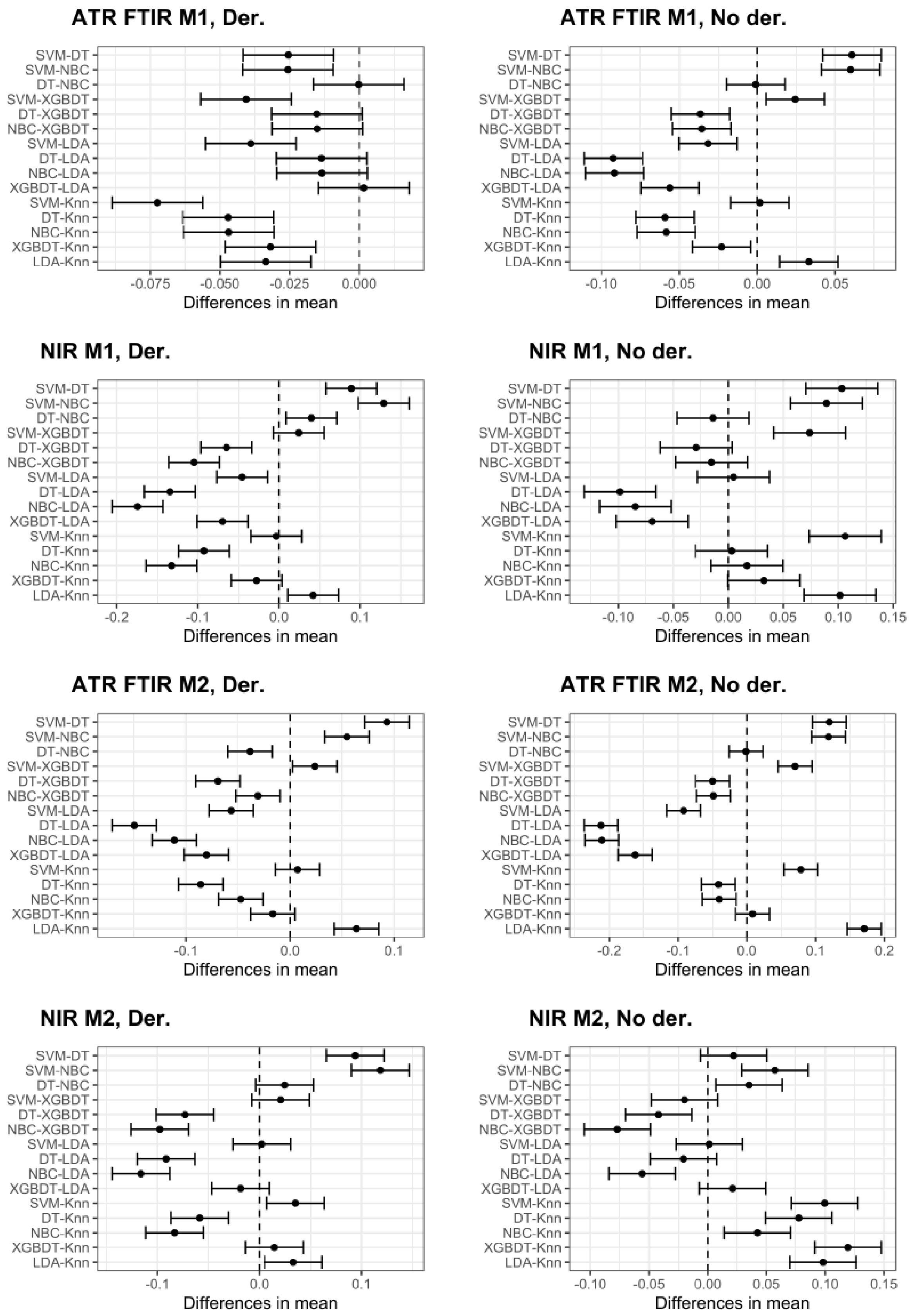

Figure S6: The pairwise comparisons for all developed classification models (95% family-wise confidence level). Confidence intervals that include 0 indicate pair of algorithms that are not significantly different ($p > 0.05$).

5.3. Confusion matrices

Table S8: Relative confusion matrices for the most accurate M1 classification models. The rows present true plasticizer type, while the columns present the predicted type. The numbers depict the relative misclassification values normalised by row/column.

M1 ATR FTIR LDA No der.

**true / predicted**	**DEHP**	**DOTP**	**none**	**-err.-**
**DEHP**	0.988/0.969	0.003/0.006	0.009/0.013	0.012
**DOTP**	0.000/0.000	0.992/0.984	0.008/0.005	0.008
**none**	0.041/0.031	0.006/0.010	0.953/0.983	0.047
**-err.-**	0.03	0.02	0.02	0.02

M1 NIR LDA Der.

**true / predicted**	**DEHP**	**DOTP**	**none**	**-err.-**
**DEHP**	0.921/0.925	0.020/0.047	0.059/0.072	0.08
**DOTP**	0.042/0.018	0.930/0.949	0.028/0.015	0.07
**none**	0.069/0.056	0.002/0.004	0.929/0.913	0.07
**-err.-**	0.07	0.05	0.09	0.07

M1 ATR FTIR kNN Der.

**true / predicted**	**DEHP**	**DOTP**	**none**	**-err.-**
**DEHP**	1.000/0.970	0.000/0.000	0.000/0.000	0.00
**DOTP**	0.000/0.000	1.000/1.000	0.000/0.000	0.00
**none**	0.040/0.030	0.000/0.000	0.960/1.000	0.04
**-err.-**	0.03	0.00	0.00	0.01

Table S9: Relative confusion matrices for the most accurate M2 models. The rows present true plasticizer type, while the columns present the predicted type. The numbers depict the relative misclassification values normalised by row/column.

M2 ATR FTIR LDA No der.

**true / predicted**	**DEHP**	**DIDP**	**DINP**	**DOTP**	**none**	**DINP + DIDP**	**-err.-**
**DEHP**	1.000/1.000	0.000/0.000	0.000/0.000	0.000/0.000	0.000/0.000	0.000/0.000	0.000
**DIDP**	0.000/0.000	1.000/1.000	0.000/0.000	0.000/0.000	0.000/0.000	0.000/0.000	0.000
**DINP**	0.000/0.000	0.000/0.000	1.000/1.000	0.000/0.000	0.000/0.000	0.000/0.000	0.000
**DOTP**	0.000/0.000	0.000/0.000	0.000/0.000	1.000/0.996	0.000/0.000	0.000/0.000	0.000
**none**	0.000/0.000	0.000/0.000	0.000/0.000	0.002/0.004	0.998/1.000	0.000/0.000	0.002
**DINP + DIDP**	0.000/0.000	0.000/0.000	0.000/0.000	0.000/0.000	0.000/0.000	1.000/1.000	0.000
**-err.-**	0.000	0.000	0.000	0.004	0.000	0.000	0.006

M2 NIR LDA Der.

**true / predicted**	**DEHP**	**DIDP**	**DINP**	**DOTP**	**none**	**DINP + DIDP**	**-err.-**
**DEHP**	0.764/0.779	0.047/0.379	0.104/0.416	0.000/0.000	0.023/0.031	0.062/0.336	0.24
**DIDP**	0.512/0.056	0.142/0.124	0.150/0.065	0.000/0.000	0.000/0.000	0.197/0.115	0.86
**DINP**	0.418/0.090	0.120/0.207	0.153/0.130	0.000/0.000	0.004/0.001	0.305/0.350	0.85
**DOTP**	0.029/0.013	0.008/0.028	0.006/0.010	0.945/0.994	0.000/0.000	0.012/0.028	0.06
**none**	0.053/0.042	0.008/0.048	0.000/0.000	0.002/0.004	0.925/0.963	0.012/0.051	0.07
**DINP + DIDP**	0.117/0.020	0.158/0.214	0.566/0.379	0.005/0.002	0.020/0.005	0.133/0.120	0.87
**-err.-**	0.221	0.876	0.870	0.006	0.037	0.880	0.27

M2 NIR SVM Der.

**true / predicted**	**DEHP**	**DIDP**	**DINP**	**DOTP**	**none**	**DINP + DIDP**	**-err.-**
**DEHP**	0.907/0.620	0.042/0.274	0.031/0.324	0.007/0.024	0.010/0.014	0.000/0.029	0.09
**DIDP**	0.393/0.034	0.482/0.386	0.125/0.159	0.000/0.000	0.000/0.000	0.000/0.000	0.52
**DINP**	0.738/0.110	0.088/0.124	0.078/0.175	0.000/0.000	0.000/0.000	0.094/0.970	0.92
**DOTP**	0.316/0.094	0.017/0.050	0.000/0.000	0.664/0.974	0.001/0.000	0.000/0.000	0.34
**none**	0.102/0.054	0.000/0.000	0.000/0.000	0.000/0.001	0.896/0.980	0.000/0.000	0.10
**DINP + DIDP**	0.670/0.085	0.136/0.164	0.178/0.340	0.000/0.000	0.015/0.004	0.000/0.000	1.00
**-err.-**	0.38	0.61	0.83	0.03	0.02	1.00	0.28

M2 NIR XGBDT Der.

**true / predicted**	**DEHP**	**DIDP**	**DINP**	**DOTP**	**none**	**DINP + DIDP**	**-err.-**
**DEHP**	0.837/0.662	0.003/0.667	0.027/0.368	0.051/0.120	0.065/0.076	0.017/0.294	0.16
**DIDP**	0.735/0.057	0.009/0.167	0.009/0.011	0.000/0.000	0.145/0.017	0.103/0.176	0.99
**DINP**	0.667/0.112	0.000/0.000	0.036/0.103	0.127/0.064	0.099/0.025	0.071/0.265	0.96
**DOTP**	0.178/0.061	0.000/0.000	0.002/0.011	0.785/0.810	0.035/0.018	0.000/0.000	0.22
**none**	0.050/0.029	0.000/0.000	0.011/0.115	0.003/0.006	0.936/0.825	0.000/0.000	0.06
**DINP + DIDP**	0.562/0.079	0.005/0.167	0.162/0.391	0.000/0.000	0.186/0.039	0.086/0.265	0.91
**-err.-**	0.34	0.83	0.90	0.19	0.17	0.74	0.29

5.4. Explained variance per principal component and wavelength plot

Mathematical investigation into the explained variance, described by each principal component per wavelength, was carried out. It was shown that this procedure could aid in the assignation of significant absorption bands for plasticizers added to PVC polymeric samples.

32 DEHP-containing and pre-processed spectra were treated with PCA. Following the procedure described by Dunn[10], the individual proportions of the explained variance per principal component and wavelength were calculated. Visualisation of such data can be observed in Supplementary Figures S8 and S9.

Supplementary Figure S6 clearly demonstrates at which wavenumbers DEHP absorbs, as the wavelengths with the highest amounts of explained variance correlate with different amounts of DEHP present in the samples. The main origin of variability is therefore exactly the amount of DEHP. Looking closely at the wavenumbers, we can identify characteristic C=O stretching at 1750 cm^{-1} along with aromatic sp^2 hydrogen bending absorptions at 700-900 cm^{-1}.

Supplementary Figure S7 presents less resolved explained variance per wavelength plots, which can be explained with the fact that NIR spectra exhibit combination and overtone absorptions that cannot explicitly be linked to just one chemical species. But such plots can be used for designating certain intervals as intervals of high DEHP absorptivity, mainly the band from 1800 to 2000 nm.

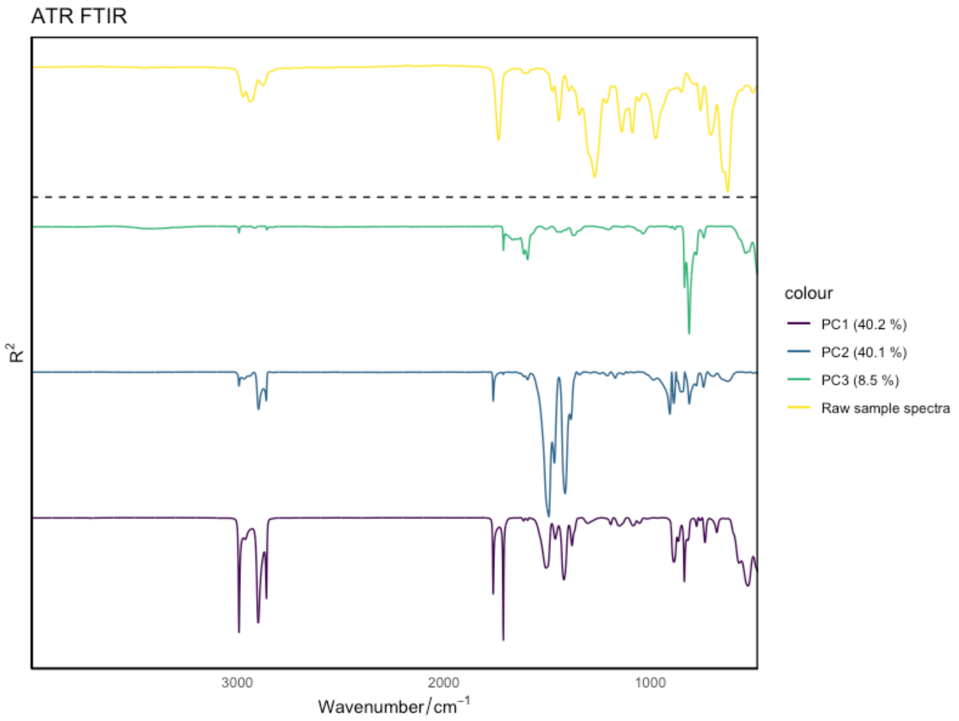

Figure S7: Explained variance per principal component and wavenumber plot for DEHP containing samples, based on ATR FTIR spectra.

[10] Dunn K (2021). "Statistics for Engineering: 6.5.11. PCA example: analysis of spectral data". https://learnche.org/pid/latent-variable-modelling/principal-component-analysis/pca-example-analysis-of-spectral-data

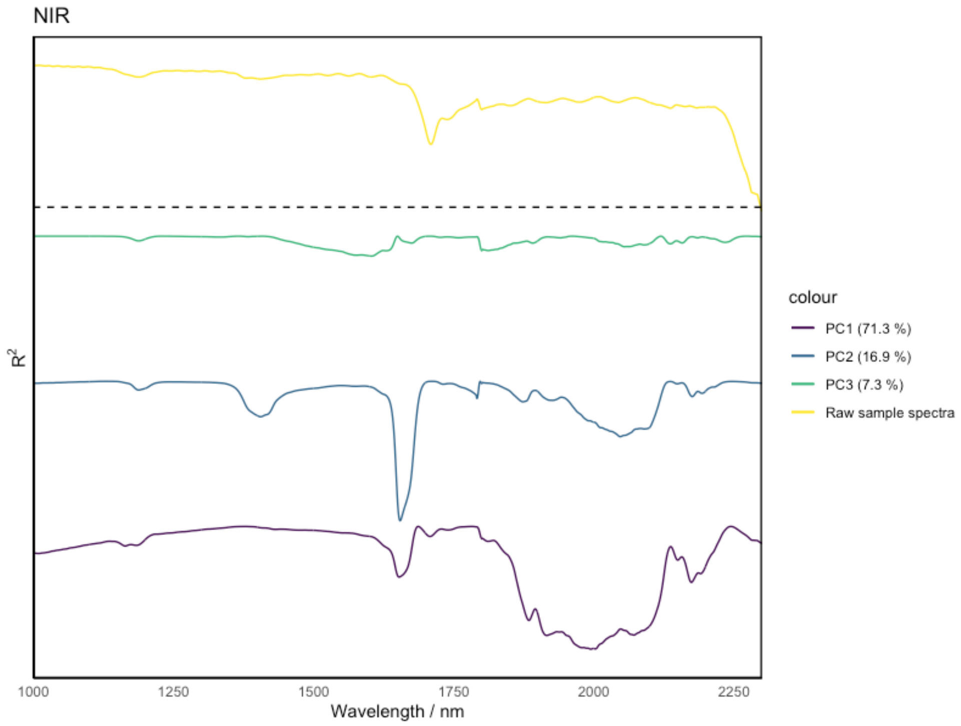

Figure S8: Explained variance per principal component and wavelength plot for DEHP containing samples, based on NIR spectra.

5.5. Regression results

Supplementary Table S10 presents detailed results of the regression models in the form of root mean squared errors of prediction and corelation coefficients along with their standard deviations.

Table S10: Results for the regression models.

	**DEHP regression model**		**DOTP regression model**	
	**R^2**	**RMSEP / %**	**R^2**	**RMSEP / %**
**ATR FTIR**	0.992 ± 0.019	0.33 ± 0.34	0.97 ± 0.11	1.4 ± 1.6
**ATR FTIR + der.**	0.9961 ± 0.0055	0.45 ± 0.21	0.970 ± 0.066	1.6± 0.94
**NIR**	0.53 ± 0.25	5.8 ± 1.2	0.033 ± 0.051	9.1 ± 2.3
**NIR + der.**	0.66 ± 0.16	5.0 ± 1.4	0.60 ± 0.32	6.2 ± 3.2

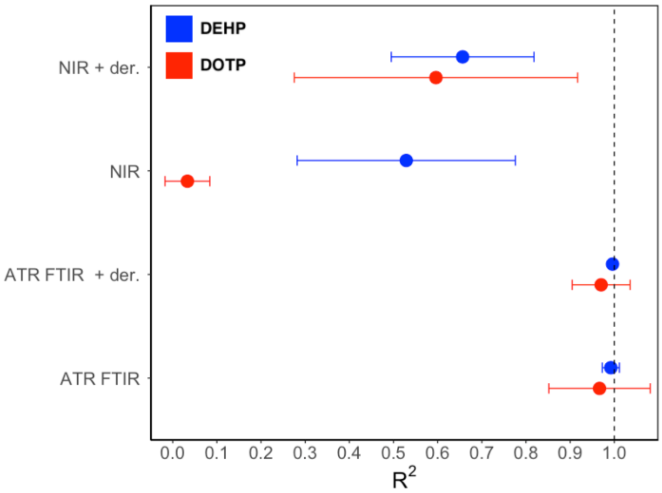

Figure S9: R^2 corelation coefficients. Blue is used to present DEHP models, while red represents DOTP models. The width of the interval presents one standard deviation.

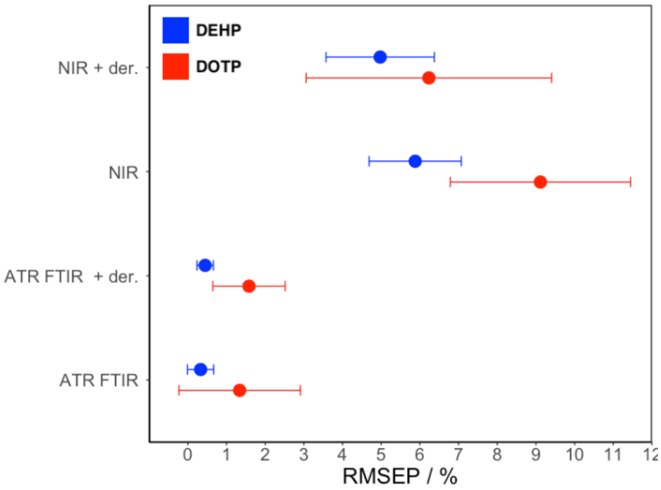

Figure S10: RMSEP values. Blue is used to present DEHP models, while red represents DOTP models. The width of the interval presents one standard deviation.
