# Supplementary material for: Machine learning-assisted non-destructive plasticizer identification and quantification in historical PVC objects based on IR spectroscopy
Source: Sci Rep. 2022 Mar 23;12:5017. doi: 10.1038/s41598-022-08862-1 (PMC8943100; doi:10.1038/s41598-022-08862-1)
Supplement: Supplementary file 1 — Supplementary Information 1. [file 41598_2022_8862_MOESM1_ESM.zip › Plasticizers_PVC_data/Supplementary_Dataset_Information.docx]

Supplementary Dataset for:

Machine learning-assisted non-destructive plasticizer identification and quantification in historical PVC objects based on IR spectroscopy

Tjaša Rijavec^1,*^, David Ribar^1^, Jernej Markelj^1^, Matija Strlič^1,2,3^, Irena Kralj Cigić^1^

*^1^Faculty of Chemistry and Chemical Technology, University of Ljubljana, Ljubljana, Slovenia*

*^2^Institute for Sustainable Heritage, University College London, London, UK*

*^3^Museum Conservation Institute, Smithsonian Institution,* *Suitland MD, USA*

*Correspondence: [tjasa.rijavec@fkkt.uni-lj.si](mailto:tjasa.rijavec@fkkt.uni-lj.si)

- Information on chromatographic plasticizer identification and quantification of 103 PVC objects available in Excel file.
- NIR spectra available in folder NIR_all.
- ATR FTIR spectra available in folder ATR_FTIR_all.
